# Body Perfusion Management in Aortic Arch Surgery

**DOI:** 10.21470/1678-9741-2024-0054

**Published:** 2025-02-15

**Authors:** Mesut Engin, Mustafa Abanoz, Ufuk Aydın, Yusuf Ata, Şenol Yavuz

**Affiliations:** 1 Department of Cardiovascular Surgery, University of Health Sciences, Bursa Yuksek Ihtisas Training and Research Hospital, Yildirim, Bursa, Turkey.; 2 Department of Cardiovascular Surgery, University of Health Sciences, Mehmet Akif Inan Training and Research Hospital, Sanliurfa, Turkey.

Dear Editor,

We have read the article by Kothari et al.^[1]^ entitled “Deep Hypothermic Cardiac Arrest Mandatory in Aortic Arch Surgeries?” with great interest. First of all, we congratulate the authors for their valuable case management. However, we would like to discuss some issues about aortic cannulation methods in aortic arch surgery (AAS).

Perfusion management is very important in AAS. In the past, total aortic arch replacement was performed with hypothermic total circulatory arrest. Today, various methods have been described in this field. In the case report by Balkanay et al.^[2]^, right axillary and femoral arterial cannulation was performed, and the “distal first technique” was applied. The authors have shown that hypothermic circulation times can be reduced with this technique. In a research article by Kiziltepe et al.^[3]^, successful applications of the left axillary artery cannulation technique was presented. The main aim of new techniques has been to reduce cardiac arrest times and circulatory arrest times.

In this current case report, the authors presented their successful AAS experience in a 16-year-old male patient. Arterial cannulation was performed through the right femoral and right axillary arteries, and cardiopulmonary bypass was provided with two-stage right atrial cannulation. The operation was performed using mild hypothermia, and equal flow was provided from both arterial lines. What was the flow rate provided to the patient during the operation? When the innominate artery and the distal part of the left subclavian artery are clamped, as shown in Figure 2, could the pressure in the arterial line be too high for the left carotid artery? Why did they use arterial circulation lines equally? Could Kiziltepe et al.'s technique be modified and preferred? We would like to receive the authors' valuable opinions on the subject.
